# Immunofluorescence labeling of cell surface antigens in *Dictyostelium*

**DOI:** 10.1186/1756-0500-6-317

**Published:** 2013-08-12

**Authors:** Alexandre Vernay, Pierre Cosson

**Affiliations:** 1 Department of Cell Physiology and Metabolism, Geneva Faculty of Medicine, Centre Médical Universitaire, 1 rue Michel Servet, Geneva 4 CH1211, Switzerland

## Abstract

**Background:**

Immunolocalization of cellular antigens typically requires fixation and permeabilization of cells, prior to incubation with antibodies.

**Findings:**

Assessing a test protein abundantly present at the cell surface of *Dictyostelium* cells, we show that in fixed cells, permeabilization extracts almost completely this cell surface antigen. The extent of this artifact is variable depending on the procedure used for labeling and permeabilization, as well as on the antigen considered.

**Conclusions:**

An optimized protocol for labeling both surface and intracellular antigens without significant loss of labeling is proposed.

## Background

In order to detect the presence of a protein in eukaryotic cells, and to determine its intracellular localization, it is common to label cells with specific fluorescent antibodies following cell fixation and permeabilization. Permeabilization must disrupt the cell membranes sufficiently to allow the passage of antibodies, while preserving the structure and protein composition of these same membranes. The problem is exacerbated at the level of the plasma membrane, which is the cellular membrane most exposed to solvents or detergents used to permeabilize cells.

*Dictyostelium discoideum* is a soil amoeba frequently used to study cell biology, in particular cell motility, endocytosis, cell adhesion or phagocytosis [1]. For many of these studies it is critical to determine if membrane proteins implicated in these processes are located in intracellular compartments or exposed at the cell surface. Protocols used to permeabilize and stain *Dictyostelium* cells are fundamentally similar to those used with mammalian cells, with the caveat that *Dictyostelium* membranes can be more resistant to mild permeabilizing detergents like saponin [2].

In the course of our studies, we observed that different immunofluorescence protocols detected very different levels of proteins at the cell surface. In this study we show that permeabilization procedures remove a large amount of cell surface antigens. We also propose an optimal procedure to label both the cell surface and intracellular compartments.

## Methods

### Cells and reagents

*Dictyostelium discoideum* DH1-10 cells [3] were grown at 21°C in HL5 medium (14.3 g/L Bactopeptone, 7.15 g/L Yeast Extract, 18 g/L Maltose monohydrate, 3.6 mM Na_2_HPO_4_.2H_2_O and 3.6 mM KH_2_PO_4_). Paraformaldehyde was purchased from by AppliChem, Saponin from Sigma and Triton X-100 was from Fluka.

The plasmid allowing expression of a fusion protein composed of the csA extracellular domain fused to the transmembrane domain of SibA and a short cytoplasmic domain (RRRSMAAA) was transfected in DH1-10 cells by electroporation. Transfected cells were then selected and grown in HL5 medium supplemented by G418 (10 μg/mL). For simplicity this fusion protein is referred to here as csA-SA. To detect csA-SA we used a mouse monoclonal antibody (41-71-21) directed to the csA extracellular domain [4]. When indicated, p23, p25 and p80 membrane proteins were detected using H194, H72, and H161 mouse monoclonal antibodies [5]. The unidentified H36 surface antigen recognized by the H36 monoclonal antibody was also described previously [6].

### Immunofluorescence

For all immunofluorescence procedures, 10^6^*Dictyostelium* cells expressing csA-SA were allowed to attach to a 22×22 mm glass coverslip for 10 minutes at room temperature in 2 mM Na_2_HPO_4_, 14.7 mM KH_2_PO_4_, pH6.0 supplemented with 0.5% HL5, 100 mM sorbitol, and 100 μM CaCl_2_. This buffer allows optimal attachment of *Dictyostelium* cells to their substrate, while preserving optimally their general organization [7]. Cells were then fixed for 10 minutes at room temperature in PBS containing 4% paraformaldehyde, then washed in PBS containing 20 mM NH_4_Cl, and in PBS containing 0.2% BSA (PBS-BSA).

In the immunofluorescence procedure referred to as “Classical”, cells were then washed twice in PBS, permeabilized in methanol at −20°C for 2 seconds, washed twice in PBS and once in PBS-BSA. When indicated, methanol was replaced with Triton X-100 (0.07% in PBS for 2 minutes at room temperature) or with saponin (0.2% in PBS for 10 minutes). Permeabilized cells were incubated with a mouse anti-csA antibody in PBS-BSA for 1 hour, washed twice in PBS-BSA, incubated for 1 hour with an Alexa-488-coupled anti-mouse immunoglobulin antibody in PBS-BSA, washed twice in PBS-BSA, once in PBS and mounted in Möwiol. Cells were visualized using a LSM700 confocal microscope (Zeiss). In each experiment, pictures from different samples were taken consecutively using identical settings.

In the procedure referred to as “Surface labeling”, non-permeabilized fixed cells were incubated with an anti-csA antibody in PBS-BSA for 1 hour, washed twice in PBS-BSA, incubated 1 hour with an Alexa-488-coupled anti-mouse antibody diluted in PBS-BSA. Finally, cells were washed twice in PBS-BSA, once in PBS and mounted in Möwiol.

In the procedure referred to as “Two-step” the surface of fixed cells was labeled as described above in the “Surface labeling” procedure. After surface labeling, cells were fixed again in paraformaldehyde, washed in PBS-NH_4_Cl, twice in PBS-BSA, twice in PBS before permeabilization in methanol at −20°C. Permeabilized cells were rinsed twice in PBS and once in PBS-BSA. Intracellular csA was then labeled for 1 hour with a mouse anti-csA antibody diluted in PBS-BSA, washed twice in PBS-BSA and revealed using an Alexa-488-coupled anti-mouse antibody. Finally, cells were washed twice in PBS-BSA, once in PBS and mounted in Möwiol.

## Findings and discussion

**Figure 1:**
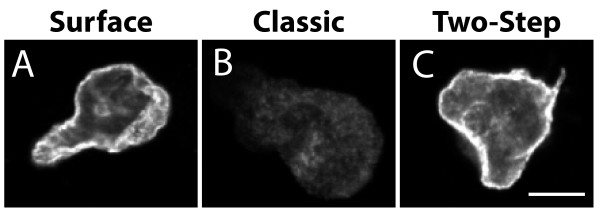
**Cell permeabilization affects detection of a surface antigen. (A)** Surface csA-SA was detected by incubating unpermeabilized fixed cells with an anti-csA antibody and a fluorescent secondary antibody. **(B)** Fixed cells were permeabilized with methanol at −20°C and then incubated sequentially with the anti-csA antibody and secondary antibodies. Surface labeling was almost entirely lost when following this classical staining procedure. **(C)** The surface of fixed cells was labeled prior to permeabilization, then cells were permeabilized and intracellular antigen stained. This two-step procedure allowed the simultaneous labeling of both surface and intracellular antigens. All the pictures presented in this figure were taken sequentially with the same microscope and with identical settings. Scale bar: 5 μm.

The csA-SA fusion protein used in this study is a single-pass type I transmembrane protein composed of the extracellular domain of the contact site A protein, fused to a single transmembrane domain and a short cytoplasmic domain. Cell surface labeling revealed that this protein was abundantly present at the surface of *Dictyostelium* cells (Figure 1A). In order to detect csA-SA both at the cell surface and in intracellular compartments, we followed a classical procedure, variations of which are most often used in many laboratories: cells were fixed with paraformaldehyde, permeabilized with methanol, then incubated sequentially with a mouse antibody against the csA moiety, and with a fluorescent secondary anti-mouse antibody. Surprisingly this procedure detected only a very small amount of protein at the cell surface (Figure 1B). This suggested that a significant amount of csA protein was lost during the procedure, particularly at the cell surface. These two procedures differ mostly by the fact that the cells are permeabilized in the latter, and previous studies have shown that a sandwich of primary and secondary antibodies can prevent loss of a cell surface protein during permeabilization [8]. Accordingly, we tested a two-step labeling procedure: cells were fixed and incubated with antibodies prior to permeabilization, then fixed again, permeabilized and intracellular antigens were labeled. This two-step procedure resulted in a prominent staining of the cell surface (Figure 1C). Together, these results indicate that the csA antigen was lost from the cell surface during cell permeabilization, unless it was stabilized by the binding of two layers of antibodies.

**Figure 2:**
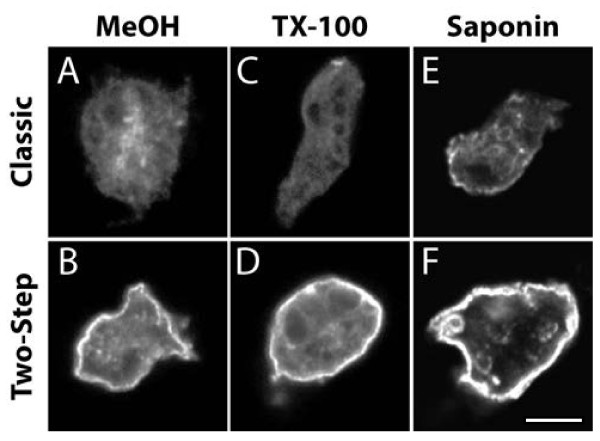
**Three distinct permeabilization procedures affect strongly detection of surface csA-SA.** Cells were treated following a classical immunofluorescence procedure (upper panel) or a two-step procedure (lower panel). Cells were permeabilized using methanol at −20°C **(A, B)** triton X-100 **(C, D)** or saponin **(E, F)**. All the pictures presented in this figure were taken sequentially with the same microscope and with identical settings. Scale bar: 5 μm.

Since methanol solubilizes and extracts cellular lipids, it may be particularly disruptive to the integrity of biological membranes. This consideration led us to test the effect of alternative permeabilization procedures. Triton X-100 is a non-ionic detergent capable of solubilizing membrane lipids. Saponin is a mild detergent extracting cholesterol from membranes and has been reported to be less prone than methanol or triton X-100 to extracting membrane proteins [9,10]. We observed that permeabilization with triton X-100 and saponin also resulted in a marked loss of surface csA-SA labeling (Figure 2C and E), although with the saponin a weak surface staining was still detectable (Figure 2E). In all cases, a two-step labeling procedure resulted in a prominent labeling of the cell surface (Figure 2B, D, F).

**Figure 3:**
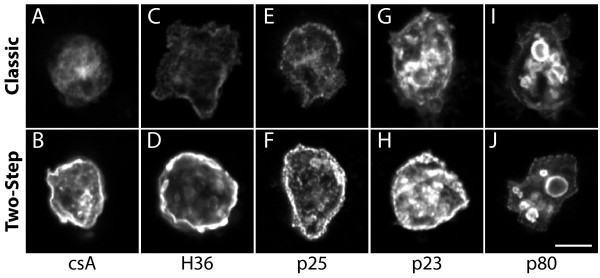
**Loss of different surface antigens during permeabilization.** Cells were labeled following a classical immunofluorescence procedure (upper panel) or a two-step procedure (lower panel). In both cases, cells were permeabilized with methanol at −20°C. The antibodies used detected csA-SA **(A, B)**, H36 **(C, D)**, p25 **(E, F)**, p23 **(G, H)** or p80 **(I, J)**. The effect of cell permeabilization on surface labeling differed for the various antigens considered. Scale bar: 5 μm.

Since the csA fusion protein analyzed in this study exhibits a single transmembrane domain and a very short cytosolic domain, it may be particularly prone to be extracted from cellular membranes. In order to study this point, we stained other membrane proteins following either a classical immunofluorescence protocol, or a two-step procedure. We used for this a collection of monoclonal antibodies recognizing antigens present at the surface of *Dictyostelium* cells [5,6]. Similar to the csA-SA protein, we observed that a classical immunofluorescence procedure resulted in a strong decrease in the cell surface labeling of the p23, p25 and H36 antigens compared to a two-step procedure (Figure 3A-H). However for these three proteins, some surface protein was still detectable even after a classical immunofluorescence staining, suggesting that they were less readily extracted from the cell surface than the csA-SA protein. The p80 protein has been shown to be a polytopic protein present at a low level at the cell surface and at a higher concentration in endosomal and lysosomal compartments [5]. The surface staining of p80 was not visibly increased by prelabeling the cell surface (Figure 3I-J), suggesting that it was not extracted from cellular membranes upon permeabilization, maybe due to the fact that this protein exhibits three transmembrane domains.

In summary, we tested here three distinct procedures to permeabilize fixed cells prior to immunofluorescence staining: methanol, triton X-100 and saponin. All three methods resulted in a marked loss of cell surface labeling of the csA-SA protein. The csA-SA protein likely represents an extreme case since it is anchored to the cell membrane only by one transmembrane domain followed by a short cytoplasmic domain. When other surface proteins were tested, some (p23, p25, H36) were also largely extracted from the cell surface although they remained detectable. On the contrary, p80, maybe due to its three transmembrane domains, was not detectably extracted from the cell surface upon permeabilization.

These results suggest that when assessing the surface localization of a protein by immunofluorescence, it is best to compare results obtained using several alternative protocols in order to ascertain that no loss of labeling is caused by the permeabilization procedure. Ideally, a surface immunofluorescence of non-permeabilized cells should be performed. In some situations, it will be difficult to detect reliably a protein of interest at the cell surface, for example if no antibodies directed to the extracellular domain of the protein are available. It may then be necessary to define the most adequate compromise to perform immunofluorescence detection: one option is to use mild detergents like saponin to reduce the amount of protein lost from the cell surface upon permeabilization. Using very low concentrations of detergents may be an alternative approach, and sufficient permeabilization may even be achieved simply by paraformaldehyde fixation with no further permeabilization [11]. It should however be kept in mind that very mild permeabilization procedures may result in incomplete permeabilization of some cellular membranes, as shown previously for saponin permeabilization in *Dictyostelium*[2]. Use of alternative methodological approaches (e.g. cell surface biotinylation followed by biochemical analysis or expression of GFP-tagged proteins in live cells) not sensitive to the same type of artifacts may be necessary to detect and quantify unambiguously the presence of a protein at the cell surface.

## Competing interests

The authors declare that they have no competing interests.

## Authors’ contributions

AV and PC contributed to the conception and the design of the study. AV performed the experiments, which were analyzed by AV and PC. AV and PC contributed to writing and final approval of the manuscript.
